# Mass Spectrometry for O-GlcNAcylation

**DOI:** 10.3389/fchem.2021.737093

**Published:** 2021-12-06

**Authors:** Ruoting Yin, Xin Wang, Cheng Li, Yuhan Gou, Xuecheng Ma, Yongzhao Liu, Jianfang Peng, Chao Wang, Ying Zhang

**Affiliations:** Key Laboratory of Resource Biology and Biotechnology in Western China, Ministry of Education, College of Life Sciences, Northwest University, Xi’an, China

**Keywords:** mass spectrometry, O-GlcNAcylation, O-GlcNAc, O-GlcNAcylated proteins, quali-quantitative charactering, isotope labeling

## Abstract

O-linked *β*-N-acetylglucosamine modification (O-GlcNAcylation) at proteins with low-abundance expression level and species diversity, shows important roles in plenty of biological processes. O-GlcNAcylations with abnormal expression levels are associated with many diseases. Systematically profiling of O-GlcNAcylation at qualitative or quantitative level is vital for their function understanding. Recently, the combination of affinity enrichment, metabolic labeling or chemical tagging with mass spectrometry (MS) have made significant contributions to structure-function mechanism elucidating of O-GlcNAcylations in organisms. Herein, this review provides a comprehensive update of MS-based methodologies for quali-quantitative characterization of O-GlcNAcylation.

## Introduction

O-GlcNAcylation, a ubiquitous post-translational modification (PTM) on nuclear, and cytoplasmic proteins (Hart et al., 2007), takes charge of numerous cardinal biological processes, such as signal transduction, transcriptional regulation, stress response, etc. Abnormal expression of O-GlcNAcylation is associated with some diseases, such as alzheimer (Yuzwa and Vocadlo, 2014), diabetes mellitus (Yang et al., 2008) and cancer (Nie et al., 2020). Therefore, the qualitative and quantitative study of glycosylation pattern of O-GlcNAcylated proteins is significant to understand the biological roles of O-GlcNAcylation during a pathological process.

Due to biological importance of O-GlcNAcylation, systematical characterization of O-GlcNAcylation has received increasing attention. However, O-GlcNAcylated proteins with multifarious types are often expressed at low level in organism, such as transcription factor CREB (Rexach et al., 2012) and protein kinase (Dias et al., 2009). Thus, systematically profiling of overall O-GlcNAcylation still faces challenges.

MS with advantages of high sensitivity and traces sample consumption has been widely used in the structural profiling of O-GlcNAcylation (Ma and Hart, 2017). Due to the low abundance and structural diversity of glycosylation, direct MS analysis of O-GlcNAcylation faces challenges. Usually, an enrichment step is necessary for MS-based profiling of O-GlcNAcylation. With the development of stable isotope tagging, quali-quantitative profiling of O-GlcNAcylation has made remarkable progress, accelerating the structure-function mechanism elucidation of O-GlcNAcylated proteins. We summarize the recent research progress in MS-based quali-quantitative analysis of O-GlcNAcylated proteins.

## Qualitative Characterization of O-GlcNAcylation by MS

### Direct MS

Earlier, collision-induced dissociation (CID), quadrupole time-of-flight (Q-TOF), electron-capture dissociation (ECD) and electron-transfer dissociation (ETD) MS have been used in O-GlcNAcylation analysis. O-GlcNAc shows easier dissociation character over other glycosylation at proteins during ionization procedure, enabling direct MS profiling of O-GlcNAc (Chalkley and Burlingame, 2001). However, the obtained GlcNAc fragment, oxonium ion, often afforded at low yield, leading to signal loss of the O-GlcNAcylation, which might be not suitable for detecting of O-GlcNAcylated proteins expressed at low levels in organism.

### Lectin Enrichment for MS

Due to the low expression level of O-GlcNAcylation, an enrichment procedure is usually needed before MS identification of the O-GlcNAcylated proteins. Lectins with feature of bonding GlcNAc have been used in enrichment of the O-GlcNAcylated proteins.

After enriching O-GlcNAcylated proteins by *Ricinus comminis agglutinin* I (RCAI) and Wheat germ agglutinin (WGA) affinity chromatography, O-GlcNAcylated proteins have been well determined by LC-ES/MS (Hayes et al., 1995; Cieniewski-Bernard et al., 2004). Succinylated wheat germ agglutinin (sWGA) and *Agrocybe aegerita lectin* 2 (AAL2), which show better binding specificity over WGA, have been used for O-GlcNAcylated proteins enrichment for subsequent MS profiling (Kupferschmid et al., 2017; Liu et al., 2018).

However, the non-specific binding of lectin to other glycan (N-glycosylated GlcNAc terminal) might decrease the detection accuracy of glycosylation. Thus, a PNGase F digestion is needed before lectin enrichment.

### Antibody Enrichment for MS

Pan-specific antibody, CTD110.6 that could bind to O-GlcNAc has been employed to enrich the O-GlcNAcylated proteins to improve MS characterization (Wells et al., 2002). To improve the enrichment of proteins, the combined utilization of three O-GlcNAc-specific IgG monoclonal antibodies [18B10.C7(3), 9D1.E4(10) and 1F5.D6(14)] to immunoprecipitate the O-GlcNAcylated proteins for subsequent O-GlcNAc-omics analysis by MS (Teo et al., 2010).

Given the importance of antibodies enrichment, the low bonding efficiency of antibodies to O-GlcNAcylated proteins and certain peptide dependence might reduce the detection accuracy.

### Metabolic Engineering and Solid Phase Enrichment for MS

**FIGURE 1:**
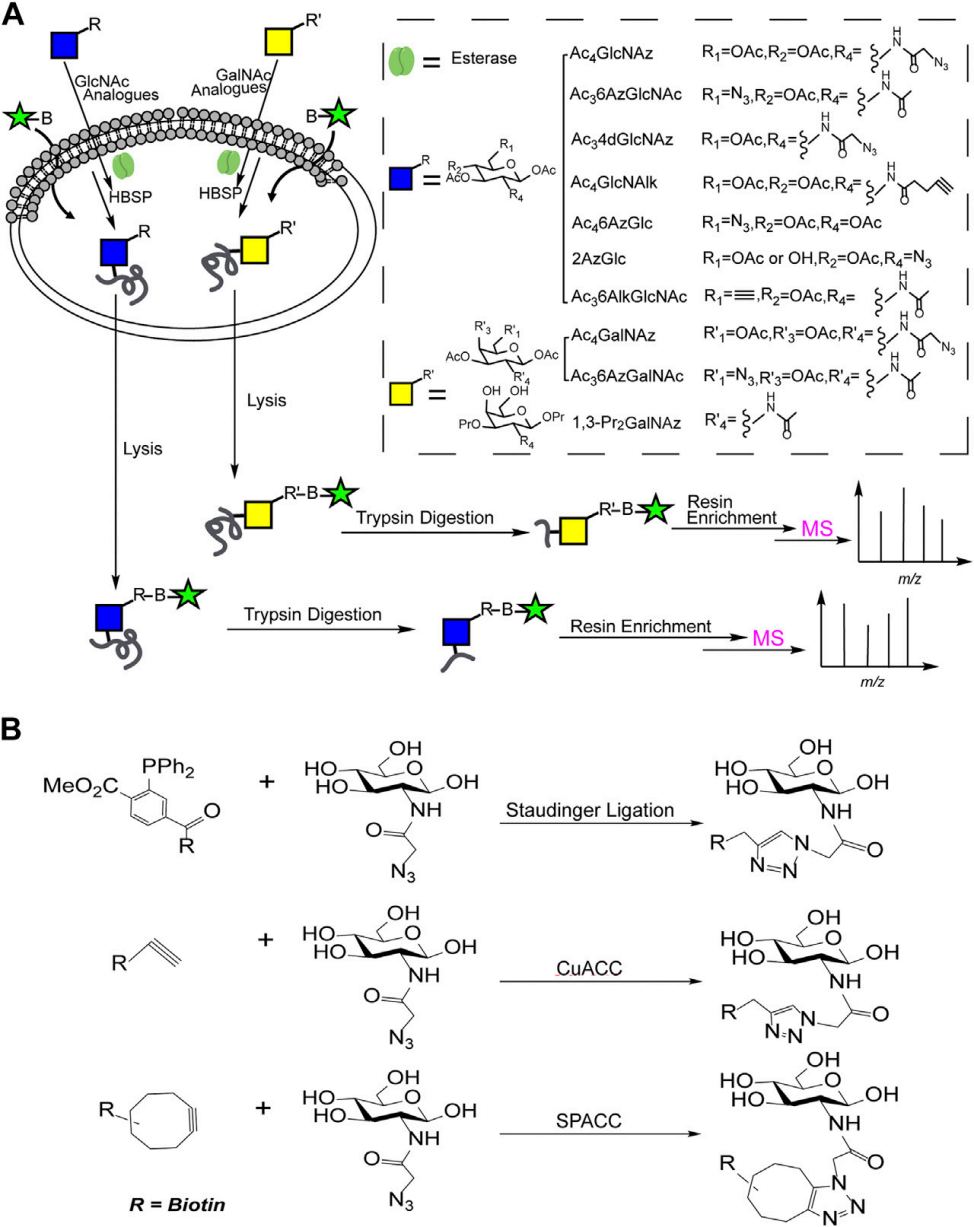
**(A)** The combination of MOE and solid phase enrichment for MS profiling of O-GlcNAcylated proteins; **(B)** Reactions involved in solid phase enrichment of O-GlcNAcylation.

With the development of metabolic oligosaccharides engineering (MOE), the O-GlcNAcylated proteins could be labeled with the reactive groups (such as alkynyl, azide, etc.) for subsequent enrichment, as shown in Figure 1. Generally, cells were cultured with metabolic chemical reporters (MCRs) such as Ac_4_GlcNAz (Sprung et al., 2005), Ac_4_GlcNAlk (Zaro et al., 2011), Ac_3_6AzGlcNAc (Chuh et al., 2014), Ac_3_4dGlcNAz (Li et al., 2016), Ac_3_6AlkGlcNAc (Chuh et al., 2017), Ac_4_6AzGlc (Darabedian et al., 2018), Ac_3_6AzGalNAc (Guo et al., 2019) and 1,3-Pr_2_GalNAz (Hao et al., 2019), etc., to synthesize O-GlcNAcylated proteins with active reactive groups. Then, the biotin probes with corresponding reactive groups (Figure 1B) were introduced to tag the labeled O-GlcNAcylations through staudinger linkage, copper-catalyzed azido-alkyne cycloaddition (CuAAC) or strain-promoted azide-alkyne cycloaddition (SPAAC). Finally, the characterization of O-GlcNAcylated proteins could be achieved by MS profiling after the biotin-avidin enrichment.

The combination of MOE and solid phase enrichment for MS profiling has made great contribution in charactering of O-GlcNAcylation. Nevertheless, some unspecific labeling to other glycosylation such as S-glycoylation was observed (Qin et al., 2020).

### Chemoenzymatic Labeling and Solid Phase Enrichment for MS

As shown in Supplementary Figure S1, GalT Y289L could transfer UDP-galactose analogues with reactive groups (ketone, alkynyl or azide) to C4-position of the O-GlcNAc at proteins. Then the labeled O-GlcNAcylated proteins could be captured by biotins with reactive groups through the orthogonal reactions such as ammoxidatin reaction (Tai et al., 2004) or click chemical reaction (Ma et al., 2019) for MS profiling. Since O-GlcNAc transferase (OGT) can recognize GlcNAc at other glycan terminals, the N-glycosylation interference should be eliminated by a PNGase F digestion before chemoenzymatic labeling.

Nevertheless, the enriched O-GlcNAcylated proteins might be difficult to elute for subsequent MS profiling. The developments of cleavable biotin linkers such as disulfide linker (Tsai et al., 2010), photocleavable linker (Li et al., 2019), acid cleavable linker (Szychowski et al., 2010), and diazobenzene linker (Yang et al., 2010), or affinity column with hydrazide cleavable linker (Nishikaze et al., 2013) to improve the dissociation efficiency of the enriched molecules have enabled more effectively profiling of O-GlcNAcylation by MS.

## Quali-Quantitative Characterization of O-GlcNAcylation by MS

### MS-Based Quali-Quantitative Characterization of O-GlcNAcylation Using ^0^D/^6^D-BEMAD Strategy

The glycosylation site of O-GlcNAcylation could be labeled with a nucleophile tag [dithiotreitol (DTT)] by *β*-elimination followed by Michael addition (BEMAD). As a result, the unstable O-GlcNAc glycosidic bond has been converted to be a stable derivative, enabling characterization of the O-GlcNAcylation by MS (Hédou et al., 2009). However, BEMAD strategy may not be suitable for distinguishing phosphorylation from O-GlcNAcylation.

When involving ^0^D/^6^D-DTT in BEMAD strategy, MS-based quali-quantitative characterization of O-GlcNAcylation could be achieved. Two samples, respectively, digested by PNGase F and trypsin digestion were subjected to ^0^D/^6^D-BEMAD, as shown in Supplementary Figure S2. Then the labeled glycopeptides captured through a mercaptans affinity chromatography and equally mixed were subjected to MS-based quali-quantitative characterization (Vosseller et al., 2005).

To improve detection efficiency and accuracy of O-GlcNAcylation by MS, an enrichment step for O-GlcNAcylated proteins has been involved (such as lectin, chemoenzyme labeling, etc.) before BEMAD.

### MS-Based Quali-Quantitative Characterization of O-GlcNAcylation by Metabolic Labeling of Stable Isotope Labels

By feeding cells with ^12^C and ^13^C glucose successively, the O-GlcNAcylated proteins could be labeled through the hexosamine biosynthetic pathway. Then the dynamic changes of O-GlcNAcylated proteins during biological procedure were determined by MS, as shown in Supplementary Figure S3A (Wang et al., 2016).

As shown in Supplementary Figure S3B, feeding cells in the presence of normal (light) or isotopically enriched (heavy) amino acid could produce normally or isotopically labeled proteins by SILAC (stable isotope labeling with amino acids). After 1:1 mixing, the mixture subjected to trypsin digestion and enriched by affinity chromatography (antibodies, lectin, etc.) to capture O-GlcNAc modified peptides, were later assigned by MS-based quali-quantitative characterization (Wang et al., 2007).

### MS-Based Quali-Quantitative Characterization of O-GlcNAcylation by Chemoenzymatic and Stable Isotope Labeling

**FIGURE 2:**
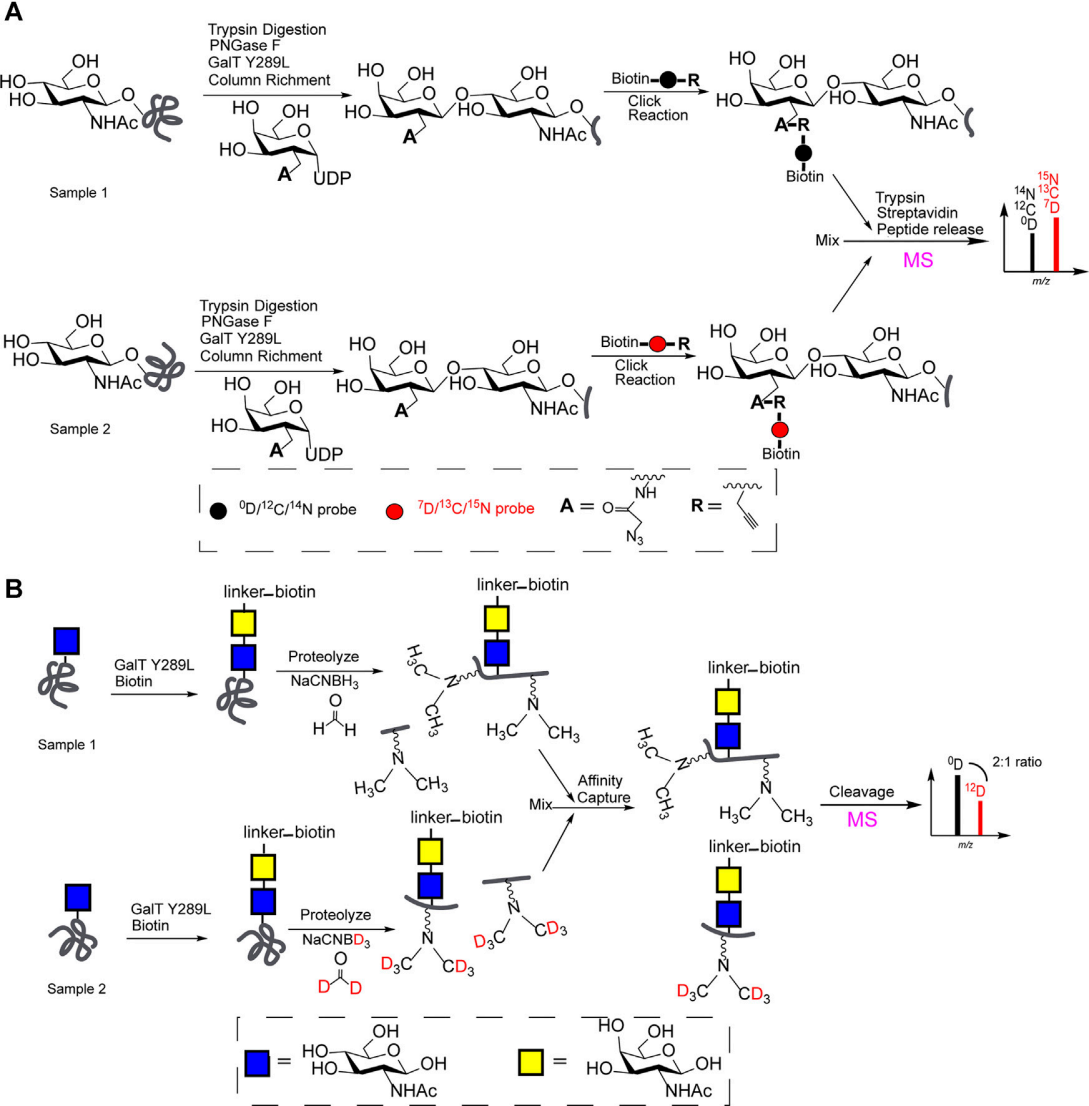
**(A)** Chemoenzymatic and stable isotope labeling (“light” and “heavy” biotin linker) for MS-based quali-quantitative characterization of O-GlcNAcylation; **(B)** QUIC-Tag for MS-based quali-quantitative profiling of O-GlcNAcylation.

As shown in Figure 2A, GalT Y289L transfers UDP-galactose analogues with reactive groups (acetylene or azide) to the C4-position of the O-GlcNAc. The “light” (^0^D, ^12^C, or ^14^N probe) and “heavy” (isotope-labeled, ^7^D, ^13^C, or ^15^N probe) biotin linker, respectively, were used to label each O-GlcNAcylation via biological orthogonal reaction. Then MS-based quali-quantitative characterization of O-GlcNAcylation between two samples was achieved after equally mixing (Qin et al., 2018; Li et al., 2019).

Meanwhile, quantitative isotopic and chemoenzymatic tagging (QUIC-Tag) for MS-based quali-quantitative profiling of O-GlcNAcylation, was illustrated in Figure 2B. Generally, samples were enriched by avidin-biotin affinity chromatography after a chemoenzymatic labeling of O-GlcNAc. After a trypsin digestion, two samples (peptides) respectively were treated with formaldehyde/NaCNBH_3_ or deuterated formaldehyde/NaCNBD_3_ *via* reductive amination reaction for subsequent MS profiling. The expression levels of O-GlcNAcylation at proteins involved in the regulation of transcription has been quantitatively characterized (Khidekel et al., 2007).

## Conclusion

O-GlcNAcylation plays an important role in plenty of biological activities, abnormal changes of O-GlcNAcylation are closely associated with the development of kinds of diseases. MS with advantages of quali-quantitatively profiling structural details of glycan compositions, glycosidic linkages and glycosylation sites, has accelerated understanding the O-GlcNAcylations.

Due to the low abundance and structural diversity of O-GlcNAc modified proteins, the combination of MOE or chemoenzymatic labeling, isotopic tagging or affinity chromatography enrichment with MS-based quali-quantitative profiling, have played important roles in understanding the biological roles of O-GlcNAcylation. However, some non-specific bonding (lectin), low bonding capacity (antibody) and unspecific labeling (S-glycosylation) occur, as summarized in Supplementary Tables S1, S2. Still, attentions should be paid to the development of specific enrichment strategy for selectively capturing the O-GlcNAcylated proteins.

Even enriching the O-GlcNAcylated proteins by specific affinity chromatography, the phosphorylation at peptide would produce the false positive signal, bringing inevitable interference in signal assignation. Efforts should be focused on developing MS-based technique combined with chemical releasing strategy to distinguish O-GlcNAcylations from O-phosphorylation in future.
