# Supplementary material for: Mass Spectrometry for O-GlcNAcylation
Source: Front Chem. 2021 Dec 6;9:737093. doi: 10.3389/fchem.2021.737093 (PMC8685217; doi:10.3389/fchem.2021.737093)
Supplement: Supplementary file 1 [file DataSheet1.doc]

# Mass Spectrometry for O-GlcNAcylation

Ruoting Yin, Xin Wang, Cheng Li, Yuhan Gou, Xuecheng Ma, Yongzhao Liu, Jianfang Peng, Chao Wang and Ying Zhang^*^

Key Laboratory of Resource Biology and Biotechnology in Western China, Ministry of Education, College of Life Sciences, Northwest University, Xi'an, 710069, P. R. China

***Correspondence:**

Ying Zhang

Email: [zhangying@nwu.edu.cn](mailto:zhangying@nwu.edu.cn)


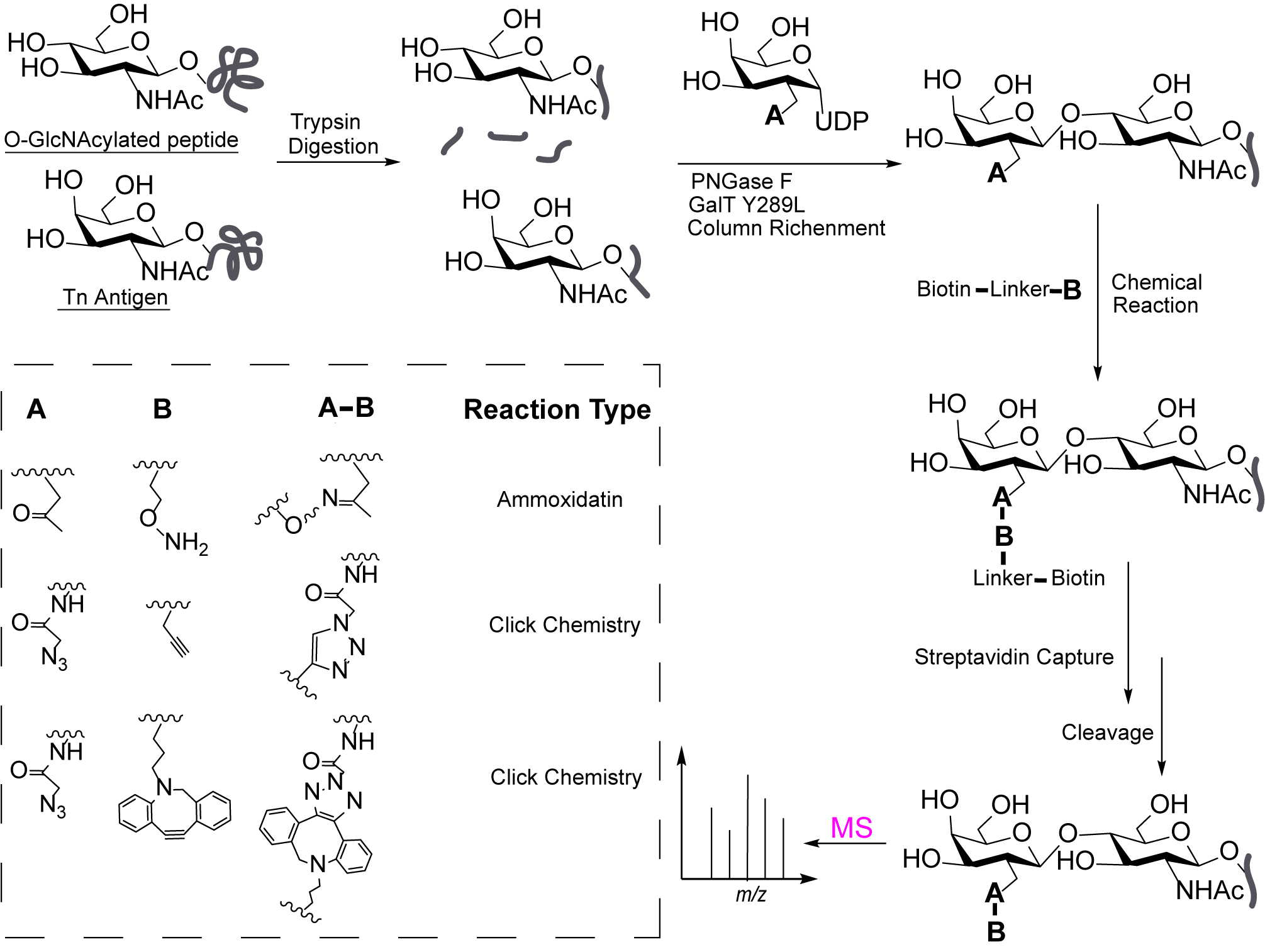


**Figure S1.** Chemoenzymatic labeling and biotin enrichment for MS-based Characterization of O-GlcNAcylation


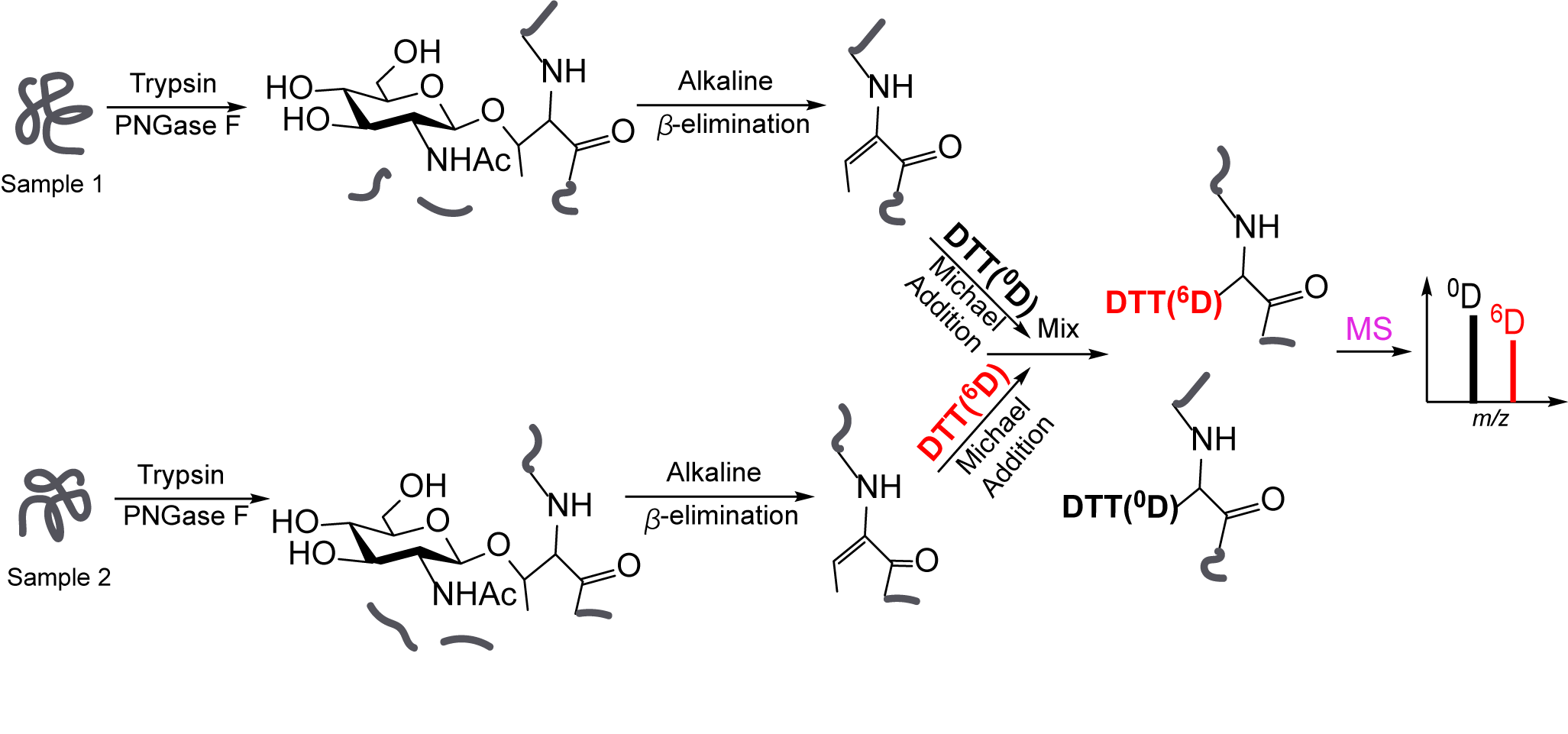


Figure S2. MS-based quali-quantitative charactering of O-GlcNAcylation using ^0^D/^6^D-BEMAD strategy


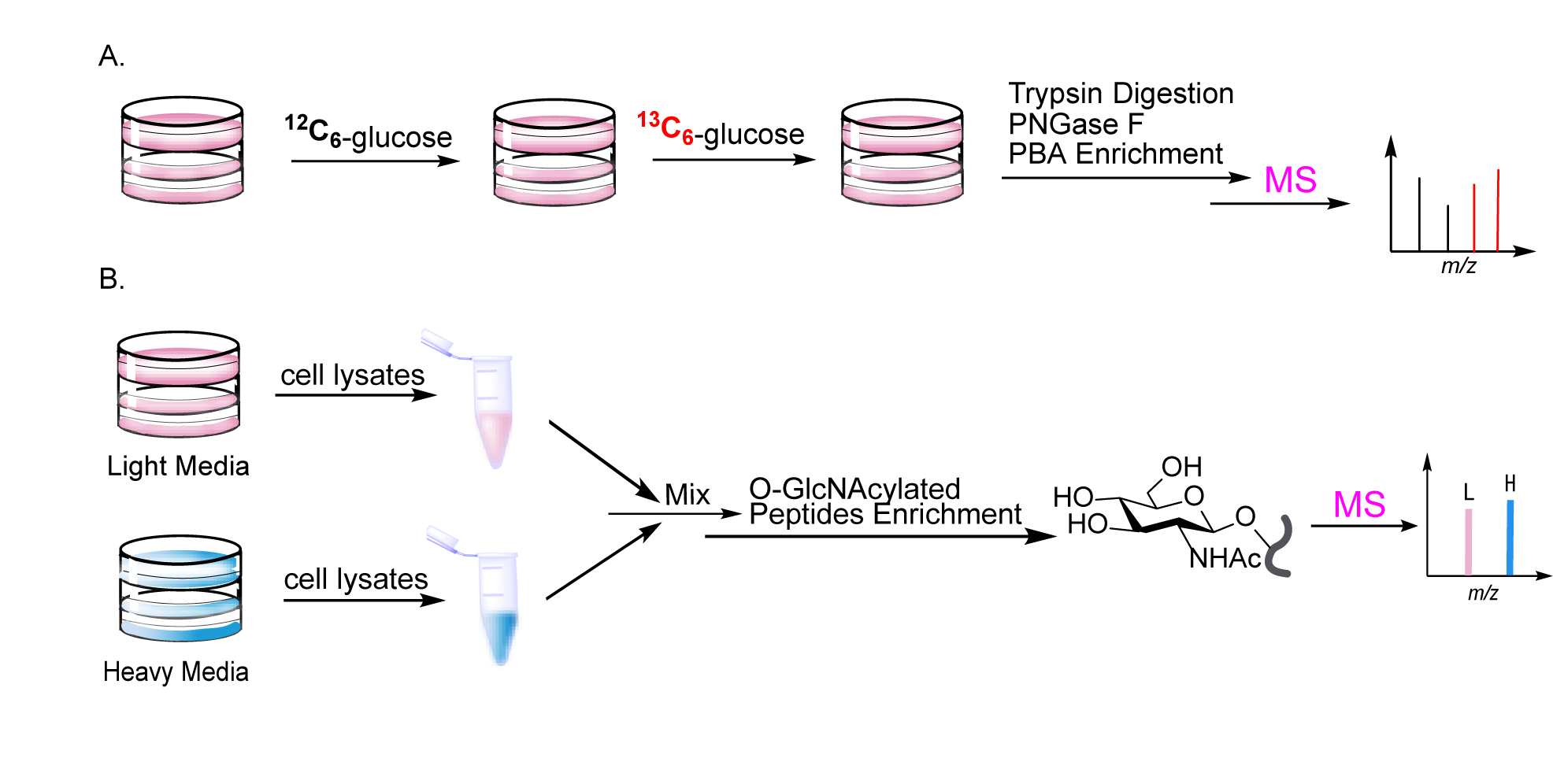


**Figure S3**. MS-based quali-quantitative charactering of O-GlcNAcylation by cell metabolism and stable isotope labeling

**Table S1. |** Comparison of MS-based Qualitative Characterization of O-GlcNAcylation

| Assay | Pros | Cons | Ref. |  |
| --- | --- | --- | --- | --- |
| **Direct MS Profiling** | Simpler | Signal loss of the O-GlcNAcylation during MS profiling | Chalkley and Burlingame, 2001 |  |
| **Lectin Enrichment for MS** | Systematically profiling | Non-specific binding of lectin to other glycan (N-glycosylated GlcNAc chain at the end) | Hayes et al., 1995; Cieniewski-Bernard et al., 2004 |  |
|  |
|  |
|  |
| **Antibody Enrichment for MS** | Systematically profiling | Low bonding efficiency of antibodies to O-GlcNAcylated proteins and certain peptide dependence | Wells et al., 2002 |  |
|  |
| **Metabolic Engineering and Solid Phase Enrichment for MS** | Systematically profiling | Some unspecific labeling to other glycosylation such as S-glycosylation was observed | Sprung et al., 2005; Zaro et al., 2011; Chuh et al., 2014; Li et al., 2016; Chuh et al., 2017; Darabedian et al., 2018; Guo et al., 2019; Hao et al., 2019 |  |
|  |
| **Chemoenzymatic Labeling and Solid Phase Enrichment for MS** | Systematically profiling | Non-specific labling of other glycan (N-glycosylated GlcNAc chain at the end) by OGT | Tai et al., 2004; Ma et al., 2019 |  |
|  |
|  |

**Table S2. |** Comparison of MS-based Quali-quantitative Characterization of O-GlcNAcylation

| Assay | Pros | Cons | Ref. |  |
| --- | --- | --- | --- | --- |
| MS-based Quali-quantitative Characterization of O-GlcNAcylation Using ^0^D/^6^D-BEMAD Strategy | Simpler | BEMAD strategy may not be suitable for distinguishing phosphorylation from O-GlcNAcylation | Hédou et al., 2009; Vosseller et al., 2005 |  |
| MS-based Quali-quantitative Characterization of O-GlcNAcylation by Metabolic Labeling of Stable Isotope Labels | Systematically profiling | Some unspecific labeling to other glycosylation such as S-glycosylation | Wang et al., 2016; Wang et al., 2007 |  |
|  |
|  |
|  |
|  |
| MS-based Quali-quantitative Characterization of O-GlcNAcylation by Chemoenzymatic and Stable Isotope Labeling | Systematically profiling | Non-specific labeling of other glycan (N-glycosylated GlcNAc chain at the end) by GalT Y289L | Qin et al., 2018; Khidekel et al., 2007 |  |
